# Thirty-Day Hospital Readmissions: A Predictor of Higher All-cause Mortality for Up to Two Years

**DOI:** 10.7759/cureus.9308

**Published:** 2020-07-21

**Authors:** Jawaid A Shaw, Spiro Stiliannoudakis, Rabia Qaiser, Erynn Layman, Adam Sima, Asghar Ali

**Affiliations:** 1 Internal Medicine, Virginia Commonwealth University, Richmond, USA; 2 Biostatistics, Virginia Commonwealth University, Richmond, USA; 3 Internal Medicine, Hunter Holmes McGuire VA Medical Center, Virginia Commonwealth University, Richmond, USA; 4 Biostatistics, Virginia Commonwealth University School of Medicine, Richmond, USA

**Keywords:** readmissions, mortality, two years, veteran affairs

## Abstract

Introduction

Readmission within 30 days is used as a standard quality metric for hospitalized patients. We hypothesized that patients who get readmitted within 30 days may have higher short- and long-term mortality.

Material and Methods

Using administrative data, we retrospectively analyzed 2,353 patients admitted to inpatient medicine service over a period of one year. The patients were matched for diagnostic group (DRG) and severity index (SI) using nearest propensity scores in a 2:1 ratio between non-readmissions (NRA) to readmissions (RA) patients.

Results

There was no statistically significant difference in the groups between age, sex, length of stay (LOS), race, and ethnicity. The hazard model yielded a hazard ratio (HR) of 2.06 for 30-day readmissions (95% CI of 1.55, 2.74; p=<0.001). The survival probability at 6, 12, 18, and 24 months was consistently greater for NRA patients.

Conclusions

Thirty-day readmissions are an independent risk factor for all-cause mortality which persists for at least two years independent of DRG and SI.

## Introduction

Readmissions within 30 days of discharge are used as a quality metric for the care of hospitalized patients. Hospitals with higher readmission rates face significant negative financial implications [1]. The magnitude of hospital readmissions is substantial and costly for the healthcare system [2]. In an ideal situation, the hospitals should be able to maintain both low rates of readmissions and mortality. On a survey of the literature, the data in regard to the direct correlation between readmissions and mortality is at best mixed, with studies revealing lower as well as higher mortality rates as discussed below [3-6]. Further on, its prognostic value has not been fully elucidated. In one study, based on individual disease states such as acute myocardial infarction, pneumonia, and congestive heart failure (CHF), it suggests that there may not be any such association [7]. In a second study, even when the rate of readmission was higher in the CHF patients the mortality rate was lower [3]. Furthermore, some existing data points toward high mortality in readmitted patients; however, most of these studies had a follow-up period of only up to a year [4-6]. These inconsistent findings generate our interest in exploring if 30-day all-cause hospital readmissions portend increased all-cause mortality in the short term and if it holds true for the future mortality as well, specifically in our veteran population. Our hypothesis being thus is that patients who get readmitted within 30 days of their discharge date may have a higher short- and long-term mortality.

## Materials and methods

This study was done using administrative data set from Hunter Holmes McGuire Veteran Affairs Medical Center, at Richmond, Virginia. After approval from the institutional review board, the electronic medical record was used for retrospective data gathering. This data comprised a total of 2,353 patients who were admitted for all causes to the internal medicine service between March 1, 2009 and February 28, 2010. Comparison of all-cause mortality between the two groups, those patients readmitted (RA) within 30 days, and those patients not getting readmitted (NRA) was made. Patients were classified as RA if they were discharged from an internal medicine service and then readmitted to an internal medicine service for any reason other than an elective procedure, within 30 days of the immediately preceding discharge. Patients were classified as NRA if they did not get readmitted to the hospital within 30 days. Only a single pair of “discharge- and-readmission/non-readmission” events was studied for each patient at the first occurrence only. Each patient was followed for a maximum of 24 months. In addition to demographic data, time to death (in days), 30-day readmission status, length of stay (LOS), diagnostic group (DRG), and severity index (SI) were recorded. Patients were categorized into one of eight different DRGs and into five groups according to SI (Table 1).

**Table 1:** Summaries and the corresponding p-values before and after using nearest neighbor propensity score matching. The means and standard deviations of each of the two groups are provided for age and length of stay, while frequencies and proportions are provided for gender, race, ethnicity, diagnostic group, and severity index (n = 2,387 for the unmatched sample; n = 735 for the matched sample).

	Unmatched Sample (n=2387)			Matched Sample (n=735)		
Covariate	Readmitted (n=245)	Not readmitted (n=2142)	P-Values	Readmitted (n=245)	Not Readmitted (n=490)	P-Values
Age (mean, sd)	67.6 (12.2)	66.4 (13.2)	0.1529	67.6 (12.2)	68.4 (13.4)	0.4211
Length of stay (mean, sd)	4.5 (4.6)	5.2 (8.7)	0.0721	4.4 (4.6)	4.5 (4.3)	0.5928
Diagnostic group			0.0019			0.9848
Cardiovascular	64 (26%)	734 (34%)		64 (26%)	118 (24%)	
Gastrointestinal	34 (14%)	236 (11%)		34 (14%)	76 (16%)	
Infections	31 (13%)	206 (10%)		31 (13%)	64 (13%)	
Metabolic	22 (9%)	157 (7%)		22 (9%)	54 (11%)	
Neurological	9 (4%)	201 (9%)		9 (4%)	17 (3%)	
Renal	17 (7%)	90 (4%)		17 (7%)	31 (6%)	
Respiratory	40 (16%)	287 (13%)		40 (16%)	76 (16%)	
Other	29 (11%)	231 (11%)		29 (11%)	54 (11%)	
Ethnicity			0.5718			0.7254
Hispanic	2 (1%)	31 (2%)		2 (1%)	6 (2%)	
Not Hispanic	243 (99%)	2,111 (98%)		243 (99%)	484 (98%)	
Gender			0.2051			0.1663
Female	6 (3%)	94 (4%)		6 (3%)	24 (3%)	
Male	239 (97%)	2,048 (96%)		239 (97%)	466 (97%)	
Race			0.6422			0.2573
White	129 (53%)	1,193 (56%)		129 (53%)	289 (59%)	
Black	113 (46%)	923 (43%)		113 (46%)	196 (40%)	
Other	3 (1%)	26 (1%)		3 (1%)	5 (1%)	
Severity Index			<0.0001			0.205
<2.5%	127 (52%)	1,468 (69%)		127 (52%)	272 (56%)	
2.5 - 5%	63 (26%)	314 (15%)		63 (26%)	143 (29%)	
5 - 10%	30 (12%)	213 (10%)		30 (12%)	39 (8%)	
10 - 30%	22 (9%)	108 (5%)		22 (9%)	32 (7%)	
>=30%	3 (1%)	39 (1%)		3 (1%)	4 (<1%)	

The Veteran Affairs Inpatient Evaluation Center (IPEC) definition of severity index in cases of non-operative patients is as follows: “predicted mortality for non-operative patients at a given location divided by the national predicted mortality for such patients.” Logistic regression modeling is used to calculate predicted mortality, including predictors such as age, marital status, comorbid illnesses, diagnoses, laboratory variables, intensive care unit admission, DRG, etc. Patients with and without readmissions were matched on DRG and SI using nearest neighbor propensity scores, estimated in a 2:1 ratio of NRA to RA. In order to evaluate the matching, the two groups were compared using t-tests and Pearson chi-square tests. A Kaplan-Meier curve was plotted against readmission status and a stratified log-rank test was performed to evaluate the overall difference in the survival probabilities of the two groups. Lastly, a stratified Cox proportional hazards model was used to estimate to model the survival distributions between RA and NRA patients while adjusting for patient and clinical characteristics including age, gender, race, ethnicity, LOS, DRG, and SI. Hazard ratios (HR) and 95% confidence intervals (CI) were reported. All statistical analyses were conducted in R version 3.4.2 (R Foundation for Statistical Computing, Vienna, Austria) at a significance level of 0.05.

## Results

**Figure 1:**
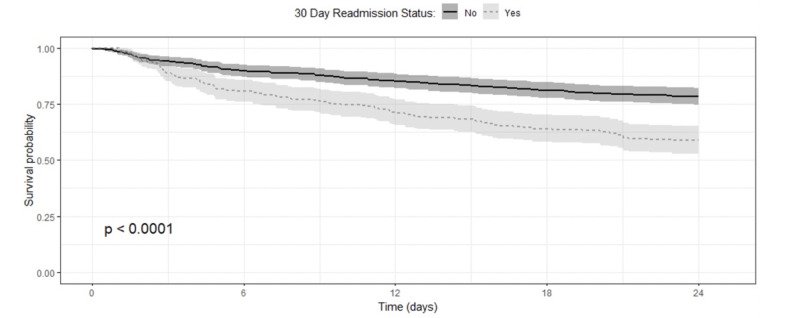
The Kaplan–Meier curves for survival following admission shows that patients that are readmitted had a higher death rate than those not readmitted in 30 days.

In our study, age, LOS, ethnicity, gender, and race were not related to readmission status, however, DRG (p=0.002) and SI (p<0.001) did have a relationship prior to matching (Table 1). Patients with cardiovascular-related diseases and SI values less than 2.5 had more readmissions. After matching, the sample size was reduced to include a total of 735 patients, 490 in NRA vs 245 in the RA group. The two groups were more balanced across the covariates of interest after matching (Table 1). The KM curves for survival following admission, separately, demonstrate that patients that are readmitted had a higher death rate than those not readmitted in 30 days (p<0.001, Figure 1).

Patients that were not readmitted within 30 days had lower mortality across the entire study. The mortality rate was consistently lower for patients that were NRA to the hospital within 30 days at 6, 12, 18, and 24 months after index admission (Table 2).

**Table 2:** Estimated mortality rates for 6, 12, 18, and 24-month time points, with corresponding 95% CI and p-values separated by readmission status.

	No readmission			Readmission			
Time (Months)	Estimated Mortality Rate	95% CI Lower Upper		EstimatedMortality Rate	95% CI Lower Upper		P-Values
6	0.102	0.075	0.128	0.192	0.859	0.141	<0.001
12	0.147	0.115	0.178	0.290	0.769	0.231	<0.001
18	0.188	0.152	0.222	0.359	0.704	0.296	<0.001
24	0.216	0.179	0.252	0.412	0.653	0.347	<0.001

The risk of death for patients that were RA to the hospital within 30 days of discharge was approximately two times that of patients that were not readmitted within 30 days (HR=2.06; 95% CI: 1.55, 2.745; p < 0.001).

## Discussion

Thirty-day readmissions were associated with increased mortality, and this risk persisted for up to two years. We matched on DRG and SI between the groups to eliminate potential bias incurred by hospital-related factors on hospital readmission. Hence, we believe that 30-day readmissions may be used as a prognosticator for poor outcomes by being able to forecast an increase in the short- and long-term mortality. 

Hospitalization has been shown to increase the risk of mortality, persisting up to a year after the index discharge in elderly patients [8]. Another study reported higher mortality (39% vs. 12%) in those who did not get readmitted within a year [5]. Our data demonstrate that there is an increased cumulative risk of mortality in patients who get readmitted within 30 days versus those not getting readmitted, which exists well up to two years. From the review of literature, one previous study which had a similar age group as in our study has shown that 30-day readmission was independently associated with higher mortality [HR=2.97] [5]. Further on, the results from two other studies suggested that 30-day readmissions are independent predictors of mortality both at 90 days and one year [6,7]. In one of the above studies, there was a significant association of 30-day readmission and mortality between those readmitted vs. not readmitted (32% vs. 14%) with an odds ratio of 2.2 [7]. The main difference between these studies and ours is that we describe differences in risk at one-year post-admission up to two years post-admission.

In the case of individual diseases, it was found that there is an association of 30-day readmissions with mortality. Arundel et al. found that in CHF patients, there was an all-cause mortality rate of 41% in readmitted patients vs. 27% in those patients who were not readmitted [HR=1.68] within one year with persistence of this association for an average of three years more [9]. Scaglione et al. reported that 50% of patients admitted for decompensated cirrhosis had 30-day readmissions resulting in higher 90-day, one-year, and overall mortality rates [10]. Nouh et al. reported a 30-day readmission rate of 8.7% in the case of stroke patients, associated with high mortality of 36.6 % vs. 14% (OR 3.6) in readmitted vs. not readmitted, but these patients were not followed up prospectively [11]. Our study is different from the above studies in that we are investigating all-cause readmissions and not those related to any single disease process, even though the theme of higher mortality rates in the readmitted patients is constant with our results.

The results of our study suggest that prevention of hospital readmissions should be a high priority to prevent increased all-cause mortality. However, there is no magical solution to reduce readmissions and a combination of approaches may be effective [12]. Various prediction models, although far from perfect, have been developed to forecast readmissions [13-14]. These could help us to prioritize our fragile inpatient resources more efficiently towards those patients who are at higher risk of getting readmitted with 30 days. Early outpatient follow-up after inpatient stay has been proven to decrease in 30-day readmissions in case of heart failure patients [15]. This correlation may hold true for other disease conditions as well. 

The limitation of our study is that it represents a single-center experience with a select cohort of veteran patients, mostly males which makes it difficult to generalize the results across all the settings. Another limitation is that the data extraction was done administratively, and some readmissions may have been missed on the electronic health record as some of these might have happened outside of the Veteran Affairs health system. We attempted to balance patient characteristics, but imbalances on unmeasured characteristics could have confounded the results. 

## Conclusions

In conclusion our study suggests that 30-day readmission regardless of overall severity of illness, is an independent risk factor for all-cause mortality which persists for at least two years. This reinforces the need for increased resource allocation to those patients who are at highest risk for readmission. This will allow for aggressive optimization of inpatient care, case management and transitions of care to prevent such readmission which will ultimately translate into decreased mortality and improved overall health-care outcomes. 
